# Supplementary material for: Meta-Analysis and Gene Set Enrichment Relative to ER Status Reveal Elevated Activity of MYC and E2F in the “Basal” Breast Cancer Subgroup
Source: PLoS One. 2009 Mar 9;4(3):e4710. doi: 10.1371/journal.pone.0004710 (PMC2650420; doi:10.1371/journal.pone.0004710)
Supplement: Dataset S1 — GSEA results from first screen. The file contains a zipped archive of the HTML results of the second GSEA screen. Reports for enriched or depleted datasets may be accessed from the “index.html” link within the my_analysis.GseaPreranked.1219975950625.rpt.zip ZIP archive. Enrichment plots are available for each of the 50 most enriched and depleted gene sets. (13.32 MB ZIP) [file pone.0004710.s001.zip › my_analysis.GseaPreranked.1219975950625.rpt.zip!//BOQUEST_CD31PLUS_VS_CD31MINUS_DN.html]

Details for gene set BOQUEST\_CD31PLUS\_VS\_CD31MINUS\_DN[GSEA]

|  || Dataset | zSco.ERneg.to.ERpos\_collapsed |
| Phenotype | NoPhenotypeAvailable |
| Upregulated in class | na\_neg |
| GeneSet | BOQUEST\_CD31PLUS\_VS\_CD31MINUS\_DN |
| Enrichment Score (ES) | -0.3277502 |
| Normalized Enrichment Score (NES) | -1.7316382 |
| Nominal p-value | 0.0 |
| FDR q-value | 0.05414213 |
| FWER p-Value | 0.935 |
Table: GSEA Results Summary

  

Fig 1: Enrichment plot: BOQUEST\_CD31PLUS\_VS\_CD31MINUS\_DN      
 Profile of the Running ES Score & Positions of GeneSet Members on the Rank Ordered List

  

| PROBE | GENE SYMBOL | GENE\_TITLE | RANK IN GENE LIST | RANK METRIC SCORE | RUNNING ES | CORE ENRICHMENT || 1 | FAT |  |  | 13 | 6.974 | 0.0119 | No |
| 2 | CRYAB |  |  | 39 | 6.465 | 0.0219 | No |
| 3 | PTX3 |  |  | 49 | 6.328 | 0.0328 | No |
| 4 | PLA2G4A |  |  | 100 | 5.860 | 0.0398 | No |
| 5 | CAPN6 |  |  | 191 | 5.356 | 0.0428 | No |
| 6 | EGFR |  |  | 344 | 4.822 | 0.0400 | No |
| 7 | DZIP1 |  |  | 393 | 4.693 | 0.0450 | No |
| 8 | ROR1 |  |  | 463 | 4.481 | 0.0480 | No |
| 9 | PDGFRA |  |  | 556 | 4.224 | 0.0487 | No |
| 10 | PCOLCE2 |  |  | 657 | 4.019 | 0.0484 | No |
| 11 | ANGPT1 |  |  | 760 | 3.824 | 0.0477 | No |
| 12 | FZD7 |  |  | 767 | 3.811 | 0.0542 | No |
| 13 | CEBPB |  |  | 795 | 3.786 | 0.0591 | No |
| 14 | MME |  |  | 901 | 3.632 | 0.0578 | No |
| 15 | SERPINE2 |  |  | 926 | 3.595 | 0.0626 | No |
| 16 | GALNT12 |  |  | 957 | 3.542 | 0.0668 | No |
| 17 | SLC39A14 |  |  | 1149 | 3.309 | 0.0583 | No |
| 18 | PLTP |  |  | 1205 | 3.229 | 0.0600 | No |
| 19 | EFS |  |  | 1238 | 3.202 | 0.0635 | No |
| 20 | S100A4 |  |  | 1324 | 3.113 | 0.0627 | No |
| 21 | DDR2 |  |  | 1334 | 3.099 | 0.0677 | No |
| 22 | OGT |  |  | 1387 | 3.052 | 0.0694 | No |
| 23 | MBP |  |  | 1428 | 3.017 | 0.0718 | No |
| 24 | CUGBP2 |  |  | 1556 | 2.898 | 0.0675 | No |
| 25 | ROBO1 |  |  | 1651 | 2.800 | 0.0654 | No |
| 26 | PLAGL1 |  |  | 1762 | 2.718 | 0.0620 | No |
| 27 | RECK |  |  | 1811 | 2.684 | 0.0633 | No |
| 28 | SCARA3 |  |  | 1813 | 2.683 | 0.0681 | No |
| 29 | LOX |  |  | 1900 | 2.605 | 0.0664 | No |
| 30 | ADCY7 |  |  | 1904 | 2.604 | 0.0709 | No |
| 31 | IGFBP3 |  |  | 1966 | 2.560 | 0.0710 | No |
| 32 | GAS1 |  |  | 1982 | 2.544 | 0.0745 | No |
| 33 | DCX |  |  | 2042 | 2.493 | 0.0746 | No |
| 34 | PRELP |  |  | 2052 | 2.482 | 0.0785 | No |
| 35 | C1R /// LOC643676 |  |  | 2173 | 2.403 | 0.0737 | No |
| 36 | LOC51334 |  |  | 2185 | 2.396 | 0.0773 | No |
| 37 | BMPR1A |  |  | 2187 | 2.394 | 0.0816 | No |
| 38 | SHOX2 |  |  | 2234 | 2.363 | 0.0824 | No |
| 39 | LIF |  |  | 2265 | 2.340 | 0.0845 | No |
| 40 | C1S |  |  | 2270 | 2.337 | 0.0885 | No |
| 41 | SLC22A3 |  |  | 2604 | 2.143 | 0.0669 | No |
| 42 | GFPT2 |  |  | 2623 | 2.133 | 0.0694 | No |
| 43 | TRO |  |  | 2765 | 2.060 | 0.0624 | No |
| 44 | SOX9 |  |  | 2768 | 2.058 | 0.0661 | No |
| 45 | AKR1C1 |  |  | 2796 | 2.041 | 0.0678 | No |
| 46 | PLP1 |  |  | 2842 | 2.019 | 0.0680 | No |
| 47 | KCNK1 |  |  | 2906 | 1.983 | 0.0669 | No |
| 48 | HDAC4 |  |  | 2922 | 1.973 | 0.0694 | No |
| 49 | CDKN2C |  |  | 3148 | 1.866 | 0.0556 | No |
| 50 | AKR1C2 |  |  | 3160 | 1.861 | 0.0582 | No |
| 51 | C6 |  |  | 3211 | 1.833 | 0.0577 | No |
| 52 | TNXB |  |  | 3236 | 1.822 | 0.0592 | No |
| 53 | FLJ20701 |  |  | 3372 | 1.761 | 0.0521 | No |
| 54 | GRIA3 |  |  | 3410 | 1.743 | 0.0525 | No |
| 55 | PEG10 |  |  | 3478 | 1.718 | 0.0505 | No |
| 56 | TMEM45A |  |  | 3492 | 1.713 | 0.0527 | No |
| 57 | COL1A1 |  |  | 3516 | 1.699 | 0.0541 | No |
| 58 | MRPS6 |  |  | 3599 | 1.664 | 0.0509 | No |
| 59 | PTGIS |  |  | 3604 | 1.661 | 0.0536 | No |
| 60 | CD44 |  |  | 3638 | 1.645 | 0.0541 | No |
| 61 | SGCE |  |  | 3724 | 1.614 | 0.0506 | No |
| 62 | MEIS2 |  |  | 3743 | 1.608 | 0.0522 | No |
| 63 | PTGFR |  |  | 3846 | 1.573 | 0.0472 | No |
| 64 | PCDH9 |  |  | 4035 | 1.499 | 0.0356 | No |
| 65 | FGF7 |  |  | 4038 | 1.499 | 0.0382 | No |
| 66 | COL14A1 |  |  | 4163 | 1.447 | 0.0314 | No |
| 67 | SART2 |  |  | 4298 | 1.393 | 0.0237 | No |
| 68 | RCN3 |  |  | 4332 | 1.379 | 0.0237 | No |
| 69 | PDE4DIP /// LOC64253 |  |  | 4470 | 1.331 | 0.0157 | No |
| 70 | PDE4DIP |  |  | 4472 | 1.330 | 0.0180 | No |
| 71 | NT5E |  |  | 4512 | 1.313 | 0.0175 | No |
| 72 | FBLN5 |  |  | 4604 | 1.285 | 0.0129 | No |
| 73 | NTRK2 |  |  | 4774 | 1.230 | 0.0022 | No |
| 74 | PPL |  |  | 4898 | 1.189 | -0.0050 | No |
| 75 | PLA2G2A |  |  | 4925 | 1.182 | -0.0049 | No |
| 76 | CHL1 |  |  | 4958 | 1.169 | -0.0052 | No |
| 77 | EMP3 |  |  | 4980 | 1.164 | -0.0046 | No |
| 78 | TNC |  |  | 5078 | 1.123 | -0.0100 | No |
| 79 | MFAP2 |  |  | 5085 | 1.121 | -0.0084 | No |
| 80 | NOTCH2 |  |  | 5101 | 1.116 | -0.0075 | No |
| 81 | RHOBTB3 |  |  | 5120 | 1.109 | -0.0068 | No |
| 82 | ANPEP |  |  | 5138 | 1.102 | -0.0061 | No |
| 83 | SDC2 |  |  | 5140 | 1.101 | -0.0041 | No |
| 84 | SLIT3 |  |  | 5203 | 1.080 | -0.0069 | No |
| 85 | F3 |  |  | 5288 | 1.053 | -0.0114 | No |
| 86 | COL6A1 |  |  | 5465 | 0.987 | -0.0230 | No |
| 87 | PTGDS |  |  | 5528 | 0.965 | -0.0260 | No |
| 88 | BHMT2 |  |  | 5544 | 0.960 | -0.0254 | No |
| 89 | FMO1 |  |  | 5595 | 0.945 | -0.0275 | No |
| 90 | WDR41 |  |  | 5682 | 0.918 | -0.0324 | No |
| 91 | MYOC |  |  | 5697 | 0.912 | -0.0318 | No |
| 92 | C3 /// LOC653879 |  |  | 5789 | 0.881 | -0.0371 | No |
| 93 | GDF10 |  |  | 5841 | 0.864 | -0.0394 | No |
| 94 | STEAP1 |  |  | 5866 | 0.855 | -0.0397 | No |
| 95 | FBLN1 |  |  | 5872 | 0.853 | -0.0385 | No |
| 96 | IRS2 |  |  | 5873 | 0.853 | -0.0369 | No |
| 97 | PTPRD |  |  | 6020 | 0.804 | -0.0466 | No |
| 98 | INHBA |  |  | 6164 | 0.762 | -0.0562 | No |
| 99 | FEZ1 |  |  | 6211 | 0.747 | -0.0583 | No |
| 100 | PDE4DIP /// LOC64254 |  |  | 6336 | 0.706 | -0.0665 | No |
| 101 | MSC |  |  | 6353 | 0.701 | -0.0664 | No |
| 102 | GPNMB |  |  | 6438 | 0.671 | -0.0716 | No |
| 103 | ANK2 |  |  | 6506 | 0.647 | -0.0756 | No |
| 104 | MEG3 |  |  | 6514 | 0.645 | -0.0749 | No |
| 105 | SSPN |  |  | 6520 | 0.643 | -0.0741 | No |
| 106 | PPP4R2 |  |  | 6673 | 0.598 | -0.0847 | No |
| 107 | ATP8B4 |  |  | 6697 | 0.588 | -0.0853 | No |
| 108 | LSP1 |  |  | 6706 | 0.584 | -0.0849 | No |
| 109 | C14ORF139 |  |  | 6793 | 0.555 | -0.0904 | No |
| 110 | HAS1 |  |  | 6794 | 0.555 | -0.0894 | No |
| 111 | FLJ22662 |  |  | 6797 | 0.553 | -0.0886 | No |
| 112 | PDLIM3 |  |  | 6847 | 0.535 | -0.0913 | No |
| 113 | FLRT2 |  |  | 6894 | 0.521 | -0.0939 | No |
| 114 | SRPX |  |  | 7014 | 0.482 | -0.1021 | No |
| 115 | UST |  |  | 7123 | 0.444 | -0.1096 | No |
| 116 | PHLDA2 |  |  | 7244 | 0.404 | -0.1180 | No |
| 117 | TNFAIP6 |  |  | 7256 | 0.399 | -0.1181 | No |
| 118 | TSPAN8 |  |  | 7315 | 0.378 | -0.1219 | No |
| 119 | TWIST1 |  |  | 7538 | 0.293 | -0.1383 | No |
| 120 | ABI3BP |  |  | 7546 | 0.291 | -0.1383 | No |
| 121 | APOE |  |  | 7604 | 0.268 | -0.1422 | No |
| 122 | ADH1A /// ADH1B /// |  |  | 7726 | 0.222 | -0.1511 | No |
| 123 | FAM69A |  |  | 7748 | 0.215 | -0.1523 | No |
| 124 | F10 |  |  | 7774 | 0.200 | -0.1538 | No |
| 125 | CFB |  |  | 7854 | 0.165 | -0.1596 | No |
| 126 | TMEM176B |  |  | 7976 | 0.114 | -0.1686 | No |
| 127 | MYH11 |  |  | 7989 | 0.109 | -0.1693 | No |
| 128 | HNMT |  |  | 8041 | 0.089 | -0.1731 | No |
| 129 | MAFB |  |  | 8124 | 0.064 | -0.1793 | No |
| 130 | MEIS1 |  |  | 8167 | 0.051 | -0.1824 | No |
| 131 | NET1 |  |  | 8194 | 0.037 | -0.1843 | No |
| 132 | ATP1A2 |  |  | 8297 | 0.002 | -0.1921 | No |
| 133 | FLJ22655 |  |  | 8402 | -0.039 | -0.2000 | No |
| 134 | FXYD1 |  |  | 8420 | -0.045 | -0.2012 | No |
| 135 | BHLHB3 |  |  | 8437 | -0.050 | -0.2024 | No |
| 136 | LOC339524 |  |  | 8534 | -0.095 | -0.2095 | No |
| 137 | CFH /// CFHR1 |  |  | 8559 | -0.106 | -0.2112 | No |
| 138 | SUSD5 |  |  | 8565 | -0.108 | -0.2114 | No |
| 139 | MRC2 |  |  | 8705 | -0.172 | -0.2217 | No |
| 140 | AXL |  |  | 8825 | -0.221 | -0.2304 | No |
| 141 | FST |  |  | 8866 | -0.240 | -0.2330 | No |
| 142 | GAS7 |  |  | 8881 | -0.247 | -0.2336 | No |
| 143 | PLN |  |  | 8924 | -0.274 | -0.2363 | No |
| 144 | ITGA8 |  |  | 8985 | -0.305 | -0.2404 | No |
| 145 | MFAP5 |  |  | 8990 | -0.307 | -0.2401 | No |
| 146 | SVEP1 |  |  | 9008 | -0.314 | -0.2408 | No |
| 147 | FIGF |  |  | 9109 | -0.366 | -0.2478 | No |
| 148 | CHRDL1 |  |  | 9142 | -0.379 | -0.2496 | No |
| 149 | CES1 |  |  | 9155 | -0.384 | -0.2498 | No |
| 150 | WNT5A |  |  | 9278 | -0.447 | -0.2583 | No |
| 151 | EDG2 |  |  | 9472 | -0.549 | -0.2721 | No |
| 152 | CCRL1 |  |  | 9478 | -0.552 | -0.2715 | No |
| 153 | IGF1 |  |  | 9639 | -0.637 | -0.2825 | No |
| 154 | MMP2 |  |  | 9732 | -0.685 | -0.2883 | No |
| 155 | MITF |  |  | 9764 | -0.703 | -0.2894 | No |
| 156 | C1ORF24 |  |  | 9785 | -0.714 | -0.2896 | No |
| 157 | GSN |  |  | 9824 | -0.739 | -0.2912 | No |
| 158 | ALDH1A1 |  |  | 9977 | -0.829 | -0.3013 | No |
| 159 | AOX1 |  |  | 9979 | -0.831 | -0.2998 | No |
| 160 | SCRN1 |  |  | 9991 | -0.836 | -0.2991 | No |
| 161 | LRP1 |  |  | 10000 | -0.845 | -0.2982 | No |
| 162 | CRISPLD2 |  |  | 10102 | -0.908 | -0.3042 | No |
| 163 | CDO1 |  |  | 10124 | -0.918 | -0.3042 | No |
| 164 | COL16A1 |  |  | 10157 | -0.934 | -0.3049 | No |
| 165 | SPON2 |  |  | 10204 | -0.961 | -0.3066 | No |
| 166 | TMED3 |  |  | 10240 | -0.983 | -0.3075 | No |
| 167 | CFH |  |  | 10249 | -0.991 | -0.3063 | No |
| 168 | SERPINF1 |  |  | 10347 | -1.059 | -0.3118 | No |
| 169 | PCOLCE |  |  | 10362 | -1.068 | -0.3109 | No |
| 170 | CD302 |  |  | 10393 | -1.087 | -0.3112 | No |
| 171 | BASP1 |  |  | 10394 | -1.088 | -0.3092 | No |
| 172 | DKFZP586H2123 |  |  | 10426 | -1.103 | -0.3095 | No |
| 173 | EFEMP1 |  |  | 10437 | -1.112 | -0.3082 | No |
| 174 | NOTCH2NL |  |  | 10540 | -1.199 | -0.3138 | No |
| 175 | COL6A2 |  |  | 10549 | -1.205 | -0.3122 | No |
| 176 | MEST |  |  | 10638 | -1.276 | -0.3166 | No |
| 177 | PTHB1 |  |  | 10665 | -1.293 | -0.3162 | No |
| 178 | DPT |  |  | 10735 | -1.341 | -0.3190 | No |
| 179 | NPY1R |  |  | 10744 | -1.349 | -0.3172 | No |
| 180 | COL5A2 |  |  | 10782 | -1.376 | -0.3175 | No |
| 181 | MGP |  |  | 10790 | -1.382 | -0.3155 | No |
| 182 | C22ORF9 |  |  | 10795 | -1.385 | -0.3132 | No |
| 183 | PTPRG |  |  | 10812 | -1.396 | -0.3119 | No |
| 184 | LAMA2 |  |  | 10896 | -1.471 | -0.3155 | No |
| 185 | C7 |  |  | 10961 | -1.538 | -0.3176 | No |
| 186 | OLFML2B |  |  | 10971 | -1.547 | -0.3154 | No |
| 187 | MXRA5 |  |  | 10993 | -1.562 | -0.3141 | No |
| 188 | GSTM5 |  |  | 11086 | -1.654 | -0.3181 | No |
| 189 | COL5A1 |  |  | 11129 | -1.688 | -0.3182 | No |
| 190 | ADH1B |  |  | 11183 | -1.731 | -0.3191 | No |
| 191 | LRP1B |  |  | 11185 | -1.733 | -0.3160 | No |
| 192 | CSPG2 |  |  | 11207 | -1.748 | -0.3144 | No |
| 193 | TIMP1 |  |  | 11230 | -1.769 | -0.3128 | No |
| 194 | TNXA /// TNXB |  |  | 11256 | -1.793 | -0.3114 | No |
| 195 | PRRX1 |  |  | 11265 | -1.799 | -0.3087 | No |
| 196 | MAN1C1 |  |  | 11380 | -1.914 | -0.3139 | No |
| 197 | CXCL12 |  |  | 11393 | -1.923 | -0.3113 | No |
| 198 | MEG3 /// LOC647251 |  |  | 11403 | -1.938 | -0.3084 | No |
| 199 | WISP2 |  |  | 11475 | -2.007 | -0.3102 | No |
| 200 | EDG2 /// LOC644923 |  |  | 11550 | -2.086 | -0.3120 | No |
| 201 | NOVA1 |  |  | 11652 | -2.168 | -0.3157 | No |
| 202 | ASPA |  |  | 11810 | -2.339 | -0.3234 | Yes |
| 203 | DCAMKL1 |  |  | 11812 | -2.342 | -0.3192 | Yes |
| 204 | GPC4 |  |  | 11878 | -2.414 | -0.3197 | Yes |
| 205 | FBN1 |  |  | 11888 | -2.424 | -0.3160 | Yes |
| 206 | MFAP4 |  |  | 11902 | -2.439 | -0.3125 | Yes |
| 207 | PDGFRL |  |  | 11926 | -2.470 | -0.3097 | Yes |
| 208 | SFRP4 |  |  | 11950 | -2.496 | -0.3068 | Yes |
| 209 | COL3A1 |  |  | 11953 | -2.497 | -0.3024 | Yes |
| 210 | THBS2 |  |  | 11978 | -2.536 | -0.2995 | Yes |
| 211 | CTSK |  |  | 12011 | -2.574 | -0.2973 | Yes |
| 212 | SLIT2 |  |  | 12017 | -2.581 | -0.2929 | Yes |
| 213 | SERPINA5 |  |  | 12084 | -2.680 | -0.2930 | Yes |
| 214 | COL1A2 |  |  | 12131 | -2.748 | -0.2915 | Yes |
| 215 | COLEC12 |  |  | 12160 | -2.792 | -0.2885 | Yes |
| 216 | ISLR |  |  | 12165 | -2.797 | -0.2836 | Yes |
| 217 | PTPN13 |  |  | 12213 | -2.856 | -0.2819 | Yes |
| 218 | CYBRD1 |  |  | 12227 | -2.884 | -0.2776 | Yes |
| 219 | DCN |  |  | 12265 | -2.925 | -0.2751 | Yes |
| 220 | IGFBP6 |  |  | 12398 | -3.098 | -0.2795 | Yes |
| 221 | ABCA8 |  |  | 12430 | -3.149 | -0.2760 | Yes |
| 222 | KCND2 |  |  | 12434 | -3.156 | -0.2705 | Yes |
| 223 | CDH11 |  |  | 12438 | -3.164 | -0.2649 | Yes |
| 224 | GLT8D2 |  |  | 12470 | -3.214 | -0.2613 | Yes |
| 225 | LUM |  |  | 12475 | -3.221 | -0.2557 | Yes |
| 226 | FAP |  |  | 12477 | -3.225 | -0.2498 | Yes |
| 227 | SEMA3C |  |  | 12485 | -3.241 | -0.2444 | Yes |
| 228 | SERPINA3 |  |  | 12498 | -3.257 | -0.2393 | Yes |
| 229 | ABCA6 |  |  | 12508 | -3.274 | -0.2340 | Yes |
| 230 | OGN |  |  | 12509 | -3.275 | -0.2279 | Yes |
| 231 | OLFML1 |  |  | 12556 | -3.342 | -0.2253 | Yes |
| 232 | DKK2 |  |  | 12563 | -3.362 | -0.2195 | Yes |
| 233 | APOD |  |  | 12582 | -3.398 | -0.2147 | Yes |
| 234 | CRIP1 |  |  | 12584 | -3.406 | -0.2085 | Yes |
| 235 | ITGBL1 |  |  | 12600 | -3.440 | -0.2033 | Yes |
| 236 | LTBP2 |  |  | 12632 | -3.492 | -0.1992 | Yes |
| 237 | COL6A3 |  |  | 12657 | -3.535 | -0.1945 | Yes |
| 238 | OLFML3 |  |  | 12677 | -3.574 | -0.1894 | Yes |
| 239 | ELOVL2 |  |  | 12695 | -3.604 | -0.1841 | Yes |
| 240 | AEBP1 |  |  | 12698 | -3.612 | -0.1776 | Yes |
| 241 | MXRA8 |  |  | 12703 | -3.626 | -0.1712 | Yes |
| 242 | GPC3 |  |  | 12707 | -3.629 | -0.1647 | Yes |
| 243 | SPOCK1 |  |  | 12719 | -3.653 | -0.1588 | Yes |
| 244 | HOXC6 |  |  | 12743 | -3.723 | -0.1537 | Yes |
| 245 | ECM2 |  |  | 12758 | -3.765 | -0.1479 | Yes |
| 246 | CFD |  |  | 12768 | -3.787 | -0.1416 | Yes |
| 247 | CXCL14 |  |  | 12915 | -4.187 | -0.1451 | Yes |
| 248 | C5ORF15 |  |  | 12937 | -4.282 | -0.1388 | Yes |
| 249 | SETBP1 |  |  | 12946 | -4.314 | -0.1314 | Yes |
| 250 | OMD |  |  | 12987 | -4.427 | -0.1263 | Yes |
| 251 | FMOD |  |  | 13009 | -4.484 | -0.1197 | Yes |
| 252 | HTRA1 |  |  | 13042 | -4.623 | -0.1136 | Yes |
| 253 | OSR2 |  |  | 13056 | -4.668 | -0.1060 | Yes |
| 254 | CILP |  |  | 13060 | -4.679 | -0.0976 | Yes |
| 255 | PDGFD |  |  | 13094 | -4.800 | -0.0913 | Yes |
| 256 | CRABP2 |  |  | 13116 | -4.890 | -0.0839 | Yes |
| 257 | AGL |  |  | 13156 | -5.073 | -0.0775 | Yes |
| 258 | SLC2A10 |  |  | 13202 | -5.387 | -0.0711 | Yes |
| 259 | ACOX2 |  |  | 13223 | -5.611 | -0.0623 | Yes |
| 260 | RNASE4 |  |  | 13230 | -5.708 | -0.0522 | Yes |
| 261 | GSTM3 |  |  | 13252 | -5.994 | -0.0428 | Yes |
| 262 | HSPB8 |  |  | 13256 | -6.029 | -0.0319 | Yes |
| 263 | UGDH |  |  | 13266 | -6.157 | -0.0212 | Yes |
| 264 | LRRC17 |  |  | 13294 | -6.494 | -0.0113 | Yes |
| 265 | IRS1 |  |  | 13313 | -7.188 | 0.0005 | Yes |
Table: GSEA details [plain text format]

  

Fig 2: BOQUEST\_CD31PLUS\_VS\_CD31MINUS\_DN: Random ES distribution      
 Gene set null distribution of ES for **BOQUEST\_CD31PLUS\_VS\_CD31MINUS\_DN**
